# Human Immunodeficiency Virus Type 1 (HIV-1) Subtype B Epidemic in Panama Is Mainly Driven by Dissemination of Country-Specific Clades

**DOI:** 10.1371/journal.pone.0095360

**Published:** 2014-04-18

**Authors:** Yaxelis Mendoza, Alexander A. Martínez, Juan Castillo Mewa, Claudia González, Claudia García-Morales, Santiago Avila-Ríos, Gustavo Reyes-Terán, Blas Armién, Juan M. Pascale, Gonzalo Bello

**Affiliations:** 1 Department of Genomics and Proteomics, Gorgas Memorial Institute for Health Studies, Panama City, Panama; 2 Department of Biotechnology, Acharya Nagarjuna University, Guntur City, Andhra Pradesh, India; 3 Department of Genetics and Molecular Biology, University of Panama, Panama City, Panama; 4 INDICASAT-AIP, City of Knowledge, Clayton, Panama City, Panama; 5 Centro de Investigación en Enfermedades Infecciosas, Instituto Nacional de Enfermedades Respiratorias, Mexico City, Mexico; 6 Laboratório de AIDS e Imunologia Molecular, Instituto Oswaldo Cruz, FIOCRUZ, Rio de Janeiro, Brazil; 7 Department of Emerging and Zoonotic Infectious Diseases, Gorgas Memorial Institute for Health Studies, Panama City, Panama; St. James School of Medicine, Anguilla

## Abstract

The Human immunodeficiency virus type-1 (HIV-1) subtype B is the most predominant clade in Central America; but information about the evolutionary history of this virus in this geographic region is scarce. In this study, we reconstructed the spatiotemporal and population dynamics of the HIV-1 subtype B epidemic in Panama. A total of 761 HIV-1 subtype B *pol* sequences obtained in Panama between 2004 and 2013 were combined with subtype B *pol* sequences from the Americas and Europe. Maximum Likelihood phylogenetic analyses revealed that HIV-1 subtype B infections in Panama derived from the dissemination of multiple founder viruses. Most Panamanian subtype B viruses (94.5%) belong to the pandemic viral strain proposed as originated in the US, whereas others (5.5%) were intermixed among non-pandemic Caribbean strains. The bulk (76.6%) of subtype B sequences from Panama grouped within 12 country-specific clades that were not detected in other Central American countries. Bayesian coalescent-based analyses suggest that most Panamanian clades probably originated between the early 1970s and the early 1980s. The root location of major Panamanian clades was traced to the most densely populated districts of Panama province. Major Panamanian clades appear to have experienced one or two periods of exponential growth of variable duration between the 1970s and the 2000s, with median growth rates from 0.2 to 0.4 year^−^^1^. Thus, the HIV-1 subtype B epidemic in Panama is driven by the expansion of local viral strains that were introduced from the Caribbean and other American countries at an early stage of the AIDS pandemic.

## Introduction

The first recognized cases of Acquired Immune Deficiency Syndrome (AIDS) in America occurred in the United States (US) in the early 1980s [1], [2]. Nowadays, around 3 million people (8.6% of the global epidemic) is living with Human Immunodeficiency Virus Type 1 (HIV-1) in the continent [3]. Approximately 150,000 subjects are currently infected with HIV-1 in Central America and HIV prevalence in the adult population (15–49 years) ranges from 0.3% in Costa Rica and Nicaragua to 1.4% in Belize [4]. Thus, the HIV-1 epidemic in Central America remains a major public health concern [4], [5].

The HIV-1 group M subtype B is the most prevalent viral clade in Central America [6]. Subtype B accounts for ≥98% of HIV infections in Costa Rica [7], El Salvador [8]–[10], Guatemala [11], Honduras [10], [12]–[16], Nicaragua [17] and Panama [18]–[20]. The most accepted model of HIV-1 subtype B origin and dispersion in the Americas suggests that the virus was first introduced from Central Africa into Haiti around 1966 (1962–1970), the virus was then disseminated from Haiti to other Caribbean islands and to the US around 1969 (1966–1972), and finally the virus migrated from US to other countries around the world [21]. Thus, we could distinguish one subtype B lineage globally disseminated (“B_PANDEMIC_” clade) and some other lineages restricted to the Caribbean region (“B_CAR_” clade) in the Americas [21], [22]. Little information is available, however, about the origin of HIV-1 subtype B circulating in Central America.

A recent study that analyzed 728 HIV-1 subtype B *pol* gene sequences from Honduras, El Salvador, Panama and Belize showed that Central American subtype B populations are genetically structured according to the country of origin and estimate the origin of those epidemics between the early and the late 1990s [23]. However, another recent phylogenetic analysis of 625 HIV-1 subtype B *pol* gene sequences from Central America identified one large monophyletic lineage (“B_CAM_” clade), which comprises ∼60% of subtype B sequences from the region, and a few additional country-specific clades of small sizes [17]. The authors conclude that a single introduction of subtype B during the 1960s accounts for most HIV-1 current cases in Central America; whereas discrete sub-epidemics were generated during the 1970s by the spread of additional founder strains within country-specific transmission networks.

Panama is the southernmost Central American country and accounts for 8% of the Central America’s population and 11% of the HIV-infected people in the region [4], [24]. It was estimated that about 18,000 subjects were living with HIV in Panama in 2011 and that HIV prevalence in the adult population (15–49 years) was around 0.7% in 2012 [4], [24]. A previous study of 133 HIV-1 subtype B *pol* sequences from Panama revealed that the majority (65.4%) of subtype B viruses from this country branched within five well supported clades that probably originated around the middle 1980s [19]. Most of the Panamanian sequences analyzed, however, were collected from one single geographic region (Panama province) over a short time interval (2004–2005). Furthermore, the mono or polyphyletic origin of Panamanian subtype B clades and their evolutionary relationship with major regional clades (B_PANDEMIC_, B_CARRIBEAN_ and B_CAM_) was not investigated.

The objective of the present study was to reconstruct the origin, spatiotemporal dynamics of dissemination and demographic history of major subtype B clades circulating in Panama and to determine their evolutionary relationships with major subtype B clades circulating in the Americas. A comprehensive data set of 629 new HIV-1 subtype B *pol* sequences from Panama generated between 2007 and 2013 was combined with published subtype B *pol* sequences from Central America, North America and the Caribbean and subjected to Maximum Likelihood and Bayesian coalescent-based analyses.

## Materials and Methods

### Ethics Statement

The study was evaluated and approved by the Gorgas Memorial Institutional Bioethics Review Board (GMIBRB). The study cohort included: 1) adults and children selected from the National Surveillance System for which the GMIBRB approved the use of samples without an informed written consent since the data were analyzed anonymously and only for epidemiological purposes; and 2) adult subjects who participated in the research project entitled “Molecular Epidemiology of HIV in the Meso-American Region” and for which informed written consent was obtained.

### HIV-1 Subtype B Panamanian Sequences

New HIV-1 subtype B *pol* sequences were obtained from 629 HIV-infected subjects from Panama, of which 61% were antiretroviral drug-experienced and 38% were drug-naïve as described in detail elsewhere [20]. Blood samples from HIV-1 seropositive individuals were collected at Gorgas Memorial Institute Clinic or received from local hospitals located in different provinces of Panama from mid-2007 to May 2013. The complete protease (PR) and the first part of the reverse transcriptase (RT) of the *pol* gene (nucleotides 2253 to 3275 of reference strain HXB2) were amplified and sequenced as previously described [20], [25]. The epidemiological information from each subject was obtained from the Drug-Resistance Genotyping Test form. These new subtype B *pol* sequences were combined with those generated during 2004–2005 from 132 AIDS and asymptomatic HIV-1-infected Panamanian individuals [19]. This resulted in a final dataset of 761 HIV-1 subtype B Panamanian sequences sampled over a time period of 10 years (2004–2013) and that were geographically distributed among the nine provinces of Panama and the native autonomous territories of Kuna Yala and Ngöbe Bugle (Fig. S1).

### HIV-1 Subtype B Reference Dataset

The HIV-1 subtype B *pol* Panamanian sequences were aligned with subtype B sequences representative of the B_PANDEMIC_ clade (US/France = 809) and the B_CAR_ clades (Caribbean = 238) (Table 1). Subtype B Panamanian sequences were also aligned with all Central American sequences (*n* = 694) that matched the selected genomic region (nucleotides 2253–3275 of reference strain HXB2). All reference sequences were available at Los Alamos HIV Sequence Database (www.hiv.lanl.gov) by April 2013 (Table 1). Only one sequence per subject was selected. The subtype assignment of all sequences included was confirmed using the REGA HIV subtyping tool v.2 [26] and by performing Maximum Likelihood (ML) phylogenetic analyses with HIV-1 group M sequences from different subtypes as described below.

**Table 1:** HIV-1 subtype B sequences.

Region	Country	New sequences	Published sequences	Sampling interval
Central America	Panama	629	132	2004–2013
	Belize	-	9	2004
	Costa Rica	-	2	2002, 2007
	El Salvador	-	170	2008–2010
	Honduras	-	513	2001–2009
Caribbean	Dominican Republic	-	78	2005–2010
	Jamaica	-	70	2005–2010
	Trinidad and Tobago	-	58	2000–2003
	Others	-	32	2000–2005
North America	United States	-	465	1982–2010
Europe	France	-	344	1983–2008

### Sequence Alignment and Phylogenetic Analysis

Sequences were aligned using the ClustalW program [27]. To avoid any bias on the phylogenetic reconstructions, all sites with major antiretroviral drug resistance mutations in PR (30, 32, 46, 47, 48, 50, 54, 76, 82, 84, 88 and 90) or RT (41, 65, 67, 69, 70, 74, 100, 101, 103, 106, 115, 138, 151, 181, 184, 188, 190, 210, 215, 219 and 230) detected in at least two sequences, were excluded from each alignment. All alignments are available from the authors upon request. Maximum Likelihood (ML) phylogenetic trees were inferred under GTR+I+Γ nucleotide substitution model selected using the jModeltest program [28]. The ML tree was reconstructed with the PhyML program [29] using an online web server [30]. Heuristic tree search was performed using the SPR branch-swapping algorithm and the reliability of the obtained topology was estimated with the approximate likelihood-ratio test (*aLRT*) [31] based on the Shimodaira-Hasegawa-like procedure. The ML trees were visualized using the FigTree v1.4.0 program [32].

### Analysis of Spatiotemporal Dispersion Pattern and Demographic History

The evolutionary rate (*µ,* nucleotide substitutions per site per year, subst./site/year), the age of the most recent common ancestor (*T*_mrca,_ years), the spatial diffusion, and the mode and rate (*r*, years^−1^) of population growth of major HIV-1 Panamanian clades were jointly estimated using the Bayesian Markov Chain Monte Carlo (MCMC) approach as implemented in BEAST v1.7.5 [33], [34]. The temporal scale of evolutionary process was directly estimated from the sampling dates of the sequences using a relaxed uncorrelated lognormal molecular clock model [35]. Migration events throughout the phylogenetic histories were reconstructed using a reversible discrete phylogeography model [36]. Changes in effective population size through time were initially estimated using a Bayesian Skyline coalescent tree prior [37] and estimates of the population growth rate were subsequently obtained using the parametric model (logistic, exponential or expansion) that provided the best fit to the demographic signal contained in datasets. Comparison between demographic models was performed using the log marginal likelihood (ML) estimation based on path sampling (PS) and stepping-stone sampling (SS) methods [38]. MCMC chains were run for 50–200×10^6^ generations. Adequate chain mixing and uncertainty in parameter estimates were assessed by calculating the effective sample size and the 95% Highest Probability Density (HPD) values respectively using the TRACER v1.5 program [39]. Maximum clade credibility (MCC) trees were summarized with TreeAnnotator v1.7.5 and visualized with FigTree v1.4.0. Migratory events were summarized using the SPREAD application [40].

### Statistical Analysis

Data were transferred from field surveillance forms to a database for statistical analyses using Epi-InfoTM Software (version 7.0.9.34; Centers for Disease Control and Prevention, Atlanta, GA) and StatsDirect statistical software v2.7.9 [41]. To assess differences in the population with known mode of sexual transmission, epidemiological and demographic characteristics were compared between each of the Panamanian HIV-1 subtype B lineages and among major clades using Chi-squared test, Chi-squared for Linear Trend test and Fisher-Freeman-Halton test for nominal independence and reported if the difference was significant at p<0.05.

### Nucleotide Sequence Accession Numbers

The new Panamanian HIV-1 subtype B sequences have been deposited in Genbank with accession numbers KJ473994 - KJ474623.

## Results

### Origin of HIV-1 Subtype Panamanian B Clades

**Figure 1:**
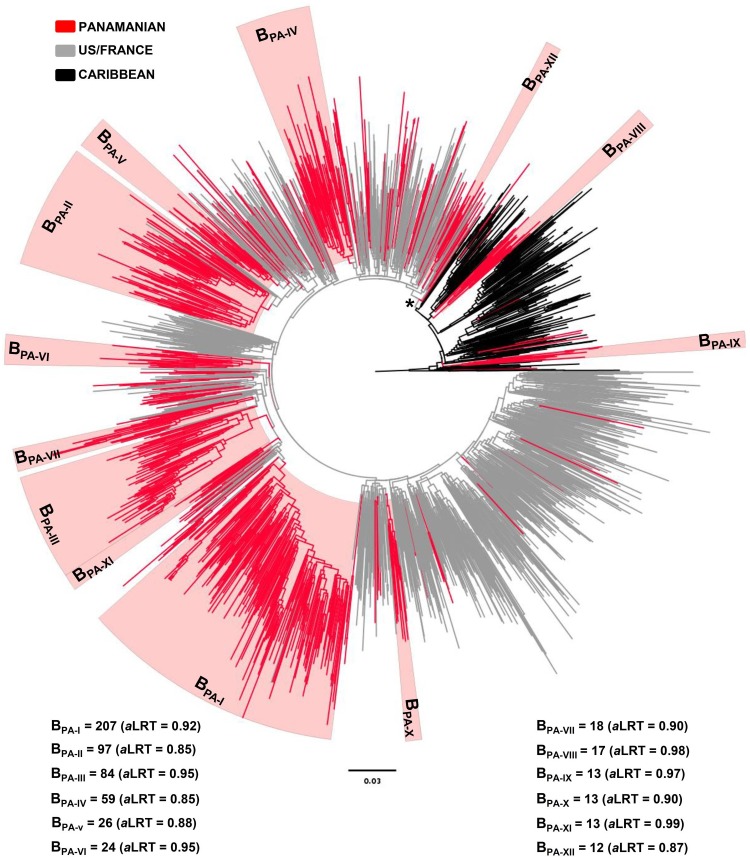
ML phylogenetic tree of HIV-1 subtype B *pol* (∼1000 pb) sequences circulating in Panama (*n* = 761), and representative sequences of the B_PANDEMIC_ (US = 465, France = 344) and the B_CAR_ (Caribbean = 238) clades. Branches are colored according to the geographic origin of each sequence, as indicated at the legend (top left). Red shaded boxes highlight the position of the twelve major Panamanian HIV-1 subtype B clades (B_PA-I_ to B_PA-XII_). The number of sequences and the *a*LRT support values for each clade are indicated at bottom. Asterisk indicates the node of the B_PANDEMIC_ clade. The tree was rooted using HIV-1 subtype D reference sequences. The branch lengths are drawn to scale with the bar at the bottom indicating nucleotide substitutions per site.

In order to better understand the origin of Panamanian HIV-1 subtype B epidemic, sequences from this country (*n* = 761) were combined with viral strains representative of the B_PANDEMIC_ (*n* = 809) and B_CAR_ (*n* = 238) lineages (Table 1). The ML analysis revealed that, as expected, sequences from Caribbean countries occupied the deepest branches within subtype B phylogeny; whereas sequences from the US and France branched in a well supported (*aLRT* = 0.89) B_PANDEMIC_ group that was nested within the B_CAR_ lineages (Fig. 1). Of the 761 Panamanian sequences analyzed, 719 (94.5%) branched within the B_PANDEMIC_ clade; whereas the remaining 42 (5.5%) were intermixed among the B_CAR_ lineages (Fig. 1). This analysis also revealed that 447 (58.7%) Panamanian sequences were distributed in four country-specific monophyletic clusters of large size (*n*>50; B_PA-I_ to B_PA-IV_), 136 (17.9%) branched in eight country-specific monophyletic clusters of medium size (10<*n*<50, B_PA-V_ to B_PA-XII_); 118 (15.5%) branched in 32 clusters of small size (2≤*n*≤10), and the remaining 60 (7.9%) represented non-clustered sequences (*n* = 1) (Fig. 1).

**Figure 2:**
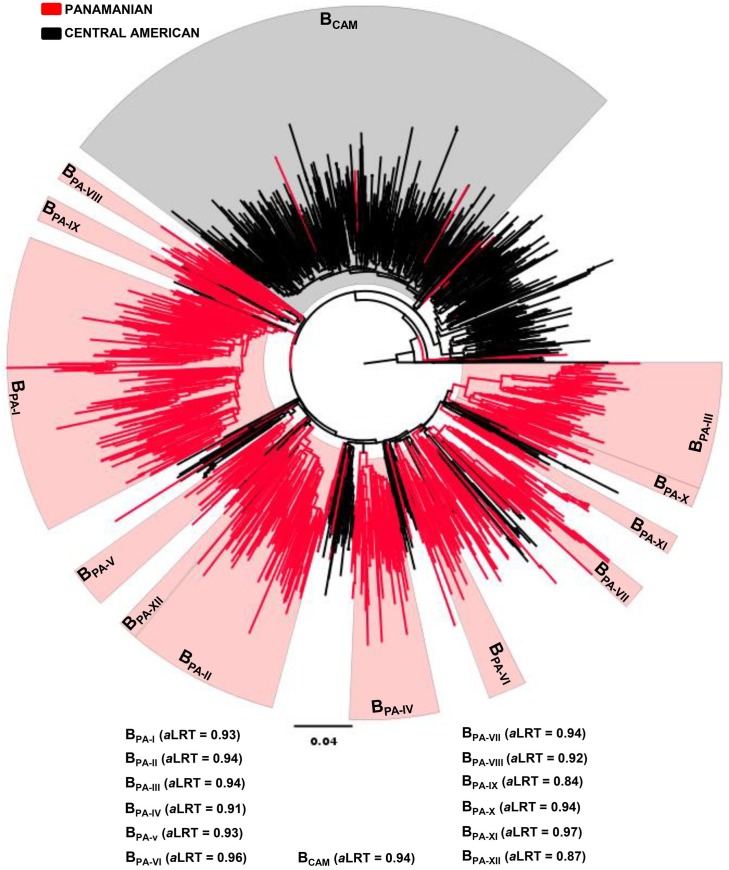
ML phylogenetic tree of HIV-1 subtype B *pol* (∼1000 pb) sequences circulating in Panama (*n* = 761) and other Central American countries (*n* = 694). The branches are colored according to the geographic origin of each sequence, as indicated at the legend (top left). Red shaded boxes highlight the position of the twelve Panamanian HIV-1 subtype B clades (B_PA-I_ to B_PA-XII_). Gray shaded box highlights the position of the major Central American clade (B_CAM_). The *a*LRT support values for each clade are indicated at bottom. The tree was rooted using HIV-1 subtype D reference sequences. The branch lengths are drawn to scale with the bar at the bottom indicating nucleotide substitutions per site.

HIV-1 subtype B Panamanian sequences were next aligned with those subtype B sequences from other Central American countries (*n* = 694). Nearly all Central American sequences available in Los Alamos database that matched the selected genomic region were from Honduras (73.9%) and El Salvador (24.5%) (Table 1). A great proportion of Central American sequences branched in a large monophyletic clade (B_CAM_, a*LRT* = 0.94) (Fig. 2), that probably corresponds to the major Central American clade recently described by Murillo *et al* (2013). The sequence composition of the clade B_CAM_ by country, however, was not homogeneous. The clade B_CAM_ comprises 71.7% of sequences from Honduras (*n* = 368), 11.1% of sequences from Belize (*n* = 1), 9.4% of sequences from El Salvador (*n* = 16) and only 0.4% (*n* = 3) of Panamanian sequences (Fig. 2). This analysis also revealed that only 0.l% (*n* = 1) of sequences from other Central American countries fell within major Panamanian clades (B_PA-I_–B_PA-XII_). This indicates a very restricted viral flow between main transmission networks from Panama and neighboring Central American countries.

### Epidemiological Characteristics of Subjects from HIV-1 Subtype B Panamanian Clades

Tables 2 and S1 summarizes the epidemiological characteristics of the present study cohort according to the main subtype B lineages and country-specific monophyletic clades that originates current Panamanian HIV epidemic. Most clades were mainly composed by male subjects with exception of clades B_PA-VIII_ and B_PA-X_. The heterosexual mode of transmission was the predominant one among clades, although clade B_PA-III_ has closer proportions of homosexual/bisexual and heterosexual modes. Diagnosis of HIV-1 infection occurred in higher frequency for year interval 2005–2009 in most clades; with exception of clade B_PA-III_ that comprises a higher frequency for year interval 2010–2013 and particularly for newly diagnosed subjects with <1 year of documented HIV-1 infection. Most subjects from medium and large clades were asymptomatic and from districts located in the east of the Panama province; except for clade B_PA-VII_ that mainly comprises subjects with AIDS diagnosis and from the province of Colon. Next, we compared the epidemiological characteristics of subjects from different clades that were infected by sexual route (Table 3). No significant differences were observed between subjects infected by the B_PANDEMIC_ and the B_CAR_ lineages; although there was a greater frequency of heterosexual transmission among B_CAR_ (96%) than among B_PANDEMIC_ (77%) infected subjects (*p* = 0.058). Comparison between major Panamanian subtype B clades (B_PA-I_ to B_PA-IV_) revealed significant differences (*p*<0.05) in the distribution of individuals according to sex and sexual mode of HIV transmission. To avoid potential bias due to the varying number of women within each clade, the variable *sexual mode of transmission* was disaggregated by gender, resulting in significant differences in the proportion of heterosexual and homosexual/bisexual males among clades (*p* = 0.041)_._ This coincides with a higher proportion of males and homosexual/bisexual men in clade B_PA-III_ (72% and 53%, respectively) than in the other major clades (35–44% and 12–18%, respectively).

**Table 2:** Epidemiological information of Panamanian subjects infected with major HIV-1 clades.

Characteristics	Total	B_CAR_	B_PANDEMIC_	B_PA-I_	B_PA-II_	B_PA-III_	B_PA-IV_
	(*n* = 629)	(*n* = 37)	(*n* = 592)	(*n* = 166)	(*n* = 74)	(*n* = 70)	(*n* = 50)
**Sampling interval (years)**	2007–2013	2009–2013	2007–2013	2007–2013	2007–2013	2008–2013	2007–2013
**HIV diagnosis**							
1987–1994	11 (1.7)	1 (2.7)	10 (1.7)	3 (1.8)	0	0	2 (4.0)
1995–1999	33 (5.2)	5 (13.5)	28 (4.7)	10 (6.0)	4 (5.4)	1 (1.4)	1 (2.0)
2000–2004	141 (22.4)	8 (21.6)	133 (22.5)	36 (21.7)	14 (18.9)	9 (12.9)	9 (18.0)
2005–2009	246 (39.1)	10 (27.0)	236 (39.9)	72 (43.4)	31 (41.9)	25 (35.7)	22 (44.0)
2010–2013	188 (30.0)	12 (32.4)	176 (29.7)	43 (25.9)	23 (31.1)	35 (50.0)	16 (32.0)
NA	10 (1.6)	1 (2.7)	9 (1.5)	2 (1.2)	2 (2.7)	0	0
**Newly diagnosed (<1 year)**	215 (34.2)	8 (21.6)	126 (21.3)	58 (34.9)	24 (32.4)	35 (50.0)	17 (34.0)
**Sex**							
Male	422 (67)	23 (62.2)	399 (67.4)	100 (60.2)	48 (64.9)	60 (85.7)	33 (66.0)
Female	207 (33)	14 (37.8)	193 (32.6)	66 (39.8)	26 (35.1)	10 (14.3)	17 (34.0)
**Mode of Transmission**							
Homosexual/Bisexual	69 (11)	1 (2.7)	68 (11.5)	12 (7.2)	7 (9.5)	19 (27.1)	3 (6.0)
Heterosexual	243 (38.6)	21 (56.8)	222 (37.5)	75 (45.2)	32 (43.2)	17 (24.3)	23 (46.0)
Mother to Child	54 (8.6)	1 (2.7)	53 (9.0)	19 (11.4)	7 (9.5)	0	1 (2.0)
Blood products	6 (0.9)	0	6 (1.0)	2 (1.2)	2 (2.7)	0	0
Unknown/NA^a^	257 (40.9)	14 (37.8)	243 (41.0)	58 (34.9)	26 (35.1)	34 (48.6)	23 (46.0)
**Age group (years)**							
<14	52 (8.3)	2 (5.4)	50 (8.4)	17 (10.2)	7 (9.5)	0	1 (2.0)
15–24	119 (18.9)	4 (10.8)	115 (19.4)	32 (19.3)	15 (20.3)	20 (28.6)	8 (16.0)
25–34	157 (25.0)	14 (37.8)	143 (24.2)	39 (23.5)	18 (24.3)	23 (32.9)	12 (24.0)
35–44	165 (26.2)	9 (24.3)	156 (26.4)	43 (25.9)	14 (18.9)	18 (25.7)	15 (30.0)
45–54	100 (15.9)	5 (13.5)	95 (16.0)	22 (13.3)	14 (18.9)	7 (10.0)	13 (26.0)
>55	36 (5.7)	3 (8.1)	33 (5.6)	13 (7.8)	6 (8.1)	2 (2.9)	1 (2.0)
**Clinical Condition**							
Asymptomatic/acute	351 (55.8)	18 (48.6)	333 (56.3)	91 (54.8)	46 (62.2)	40 (57.1)	24 (48.0)
AIDS	168 (26.7)	14 (37.8)	154 (26.0)	45 (27.1)	16 (21.6)	16 (22.9)	18 (36.0)
NA	110 (17.5)	5 (13.5)	105 (17.7)	30 (18.1)	12 (16.2)	14 (20.0)	8 (16.0)
**Geographic location**							
East of Panama Province	409 (65)	20 (54.1)	389 (65.7)	117 (70.5)	50 (67.6)	52 (74.3)	28 (56.0)
West of Panama Province	96 (15.3)	9 (24.3)	87 (14.7)	21 (12.7)	14 (18.9)	15 (21.4)	14 (28.0)
Colon Province	58 (9.2)	3 (8.1)	55 (9.3)	12 (7.2)	5 (6.8)	0	6 (12.0)
Others provinces	59 (9.4)	3 (8.1)	56 (9.5)	15 (9.0)	4 (5.4)	3 (4.3)	2 (4.0)
NA	7 (1.1)	2 (5.4)	5 (0.8)	1 (0.6)	1 (1.4)	0	0

Data are: number (%).

a Unknown/NA: subjects decide not to answer about their mode of infection or information was not available.

**Table 3:** Epidemiological data analysis of Panamanian subjects infected with major HIV-1 clades by sexual route.

Characteristics	Total	B_CAR_	B_PANDEMIC_		B_PA-I_	B_PA-II_	B_PA-III_	B_PA-IV_	
	(*n* = 312)	(*n* = 22)	(*n* = 290)	*P* value^a^	(*n* = 87)	(*n* = 39)	(*n* = 36)	(*n* = 26)	*P* value^b^
**HIV diagnosis**									
1987–2004	81 (26.0)	8 (36.4)	73 (25.2)		18 (20.7)	9 (23.1)	7 (19.4)	6 (23.1)	
2005–2009	101 (32.4)	4 (18.2)	97 (33.4)		39 (44.8)	12 (30.8)	7 (19.4)	7 (26.9)	
2010–2013	127 (40.7)	10 (45.5)	117 (40.3)	0.7266	30 (34.5)	17 (43.6)	22 (61.1)	13 (50.0)	0.1172
**HIV diagnosis**									
Newly diagnosed (<1 year)	124 (39.7)	7 (31.8)	117 (40.3)		36 (41.4)	13 (33.3)	22 (61.1)	13 (50.0)	
Previously diagnosed	185 (59.3)	15 (68.2)	170 (58.6)	0.5488	51 (58.6)	25 (64.1)	14 (38.9)	13 (50.0)	0.1005
**Sex**									
Female	179 (57.4)	14 (63.6)	165 (56.9)		57 (65.5)	22 (56.4)	10 (27.8)	17 (65.4)	
Male	133 (42.6)	8 (36.4)	125 (43.1)	0.6945	30 (34.5)	17 (43.6)	26 (72.2)	9 (34.6)	**0.0012**
**Sexual Mode of Transmission**									
Homosexual/Bisexual	69 (22.1)	1 (4.5)	68 (23.4)		12 (13.8)	7 (17.9)	19 (52.8)	3 (11.5)	
Heterosexual	243 (77.9)	21 (95.5)	222 (76.6)	0.0575	75 (86.2)	32 (82.1)	17 (47.2)	23 (88.5)	**0.0001**
**Age group (years)**									
14–24	65 (20.8)	4 (18.2)	61 (21.0)		19 (21.8)	7 (17.9)	11 (30.6)	4 (15.4)	
25–44	170 (54.5)	11 (50.0)	159 (54.8)		46 (52.9)	21 (53.8)	22 (61.1)	14 (53.8)	
45–68	77 (24.7)	7 (31.8)	70 (24.1)	0.4795	22 (25.3)	11 (28.2)	3 (8.3)	8 (30.8)	0.2733
**Clinical Condition**									
Asymptomatic	221 (70.8)	13 (59.1)	208 (71.7)		62 (71.3)	31 (79.5)	27 (75.0)	18 (69.2)	
AIDS	63 (20.2)	6 (27.3)	57 (19.7)	0.3886	17 (19.5)	4 (10.3)	7 (19.4)	5 (19.2)	0.6219
**Geographic location**									
East districts of Panama Province	214 (69.0)	12 (57.1)	202 (69.9)		64 (73.6)	29 (74.4)	27 (75.0)	16 (61.5)	
West districts of Panama Province	43 (13.9)	2 (9.5)	41 (14.2)		14 (16.1)	8 (20.5)	7 (19.4)	4 (15.4)	
Other provinces	53 (17.1)	7 (33.3)	46 (15.9)	0,0826	9 (10.3)	2 (5.1)	2 (5.6)	6 (23.1)	0.4152

Data are number (%).

a Chi-squared test or Chi-squared Trend test.

b Fisher-Freeman-Halton Test. Significant differences (*p*<0.05) are marked in bold.

### Time Scale of HIV-1 Subtype B Panamanian Clades

**Figure 3:**
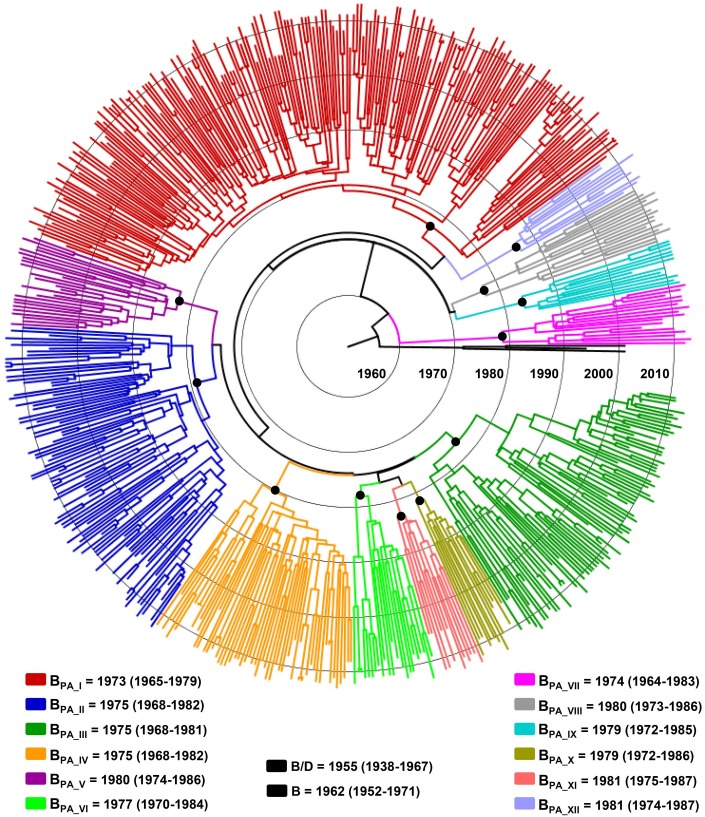
Time-scaled Bayesian MCMC tree of the major HIV-1 subtype B Panamanian clades (B_PA-I_ to B_PA-XII_) and reference subtype D sequences. Branches are colored according to the clade classification as indicated in the legend. Black key circles indicate the positions of nodes corresponding to the MCRA of each clade. The median T_MRCA_ (with the corresponding 95% credibility interval in parenthesis) of each clade is indicated at bottom. Branch lengths are depicted in units of time (years). The tree was automatically rooted under the assumption of a relaxed molecular clock.

The major HIV-1 subtype B Panamanian clades were combined in a single dataset (B_PA-I–_B_PA-XII_) and their time-scales were reconstructed under a relaxed molecular clock model using a Bayesian MCMC analysis. The different HIV-1 Panamanian clades formed highly supported monophyletic groups (*Posterior Probability* [*PP*]>0.90) in the Bayesian MCCT (Fig. 3), consistent with the ML tree topology. The median estimated evolutionary rates for this combined HIV-1 *pol* dataset was 1.9×10^−3^ (95% HPD: 1.6×10^−3^–2.3×10^−3^) subst./site/year and the coefficient of rate variation was higher than zero, thus supporting the use of a relaxed molecular clock model. Of note, the median T_MRCA_ of subtypes B/D and subtype B here estimated (1955 and 1962, respectively) were fully consistent with those described by Gilbert *et al* (2007) (1954 and 1966, respectively) (Fig. 3). This analysis indicates that subtype B Panamanian clades of large size (B_PA-I_–B_PA-IV_) probably emerged between the early and the middle 1970s; whereas, most Panamanian clades of medium size (B_PA-V_–B_PA-XII_) arise between the late 1970s and the early 1980s (Fig. 3).

### Spatial Dissemination of Major HIV-1 Subtype B Panamanian Clades

**Figure 4:**
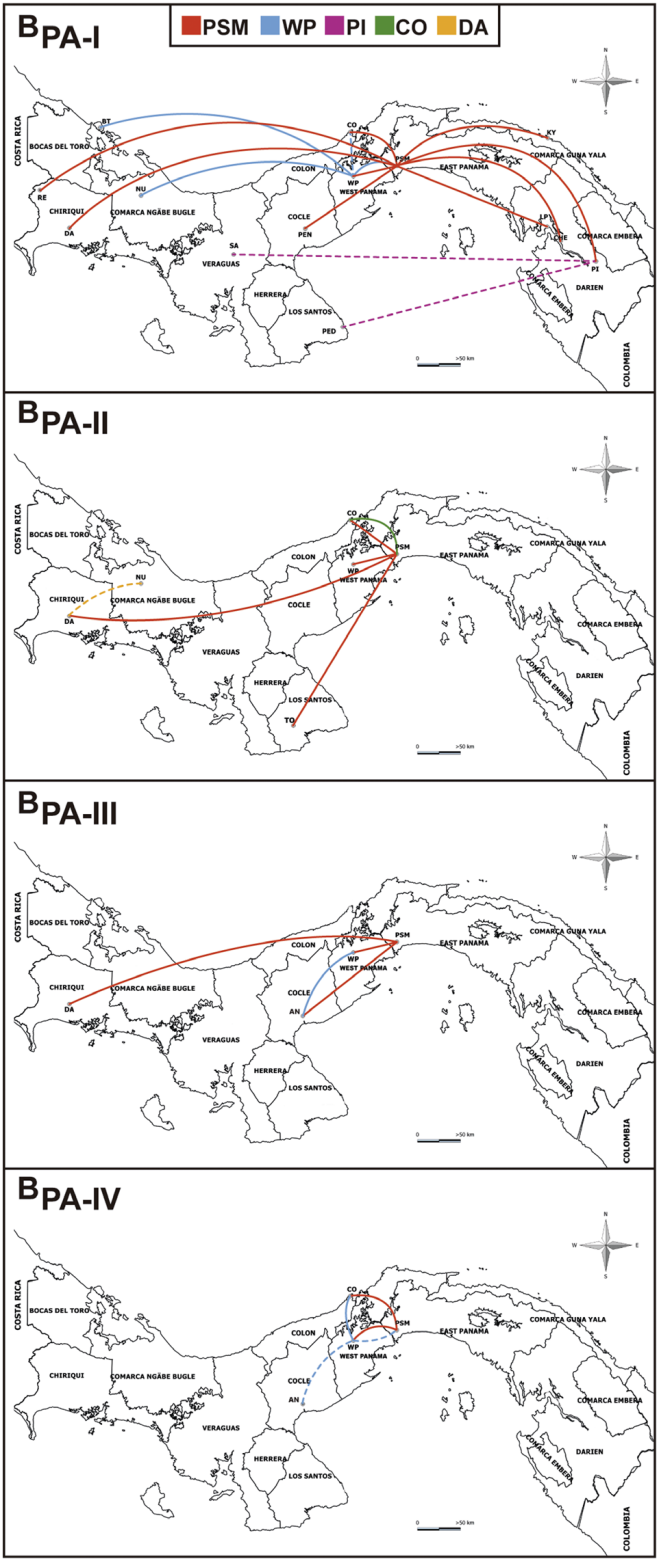
Spatial dissemination of HIV-1 Panamanian clades of large size (B_PA-I_ to B_PA-IV_) across country districts. Lines between districts represent branches in the Bayesian MCC tree along which location transitions occur. Lines were colored according to the source location as indicated in the legend at top. Dashed lines indicate those location transitions associated to nodes with low location probability support (<0.60). AN, Anton; BT, Bocas del Toro; CHE, Chepigana; CO, Colon; DA, David; KY, Kuna Yala; MU, Muna; NU, Nurum; PED, Pedasí; PEN, Penonome; PI, Pinogana; PSM, Panama and San Miguelito (east Panama province); RE, Renacimiento; SA, Santiago; TO, Tonosí; WP, west Panama province.

The spatial dissemination of HIV-1 Panamanian clades with a minimum size of 30 sequences (B_PA-I_ to B_PA-IV_) was reconstructed by analyzing each clade separately. The median evolutionary rates estimated for the different subtype B Panamanian clades were roughly similar among each other and comparable to the rate estimated for the combined dataset, thus indicating that all Panamanian clades evolved at quite similar rates (Table S2). The phylogeographic analysis showed that B_PA-I_ was the most widely disseminated lineage being detected in nearly all country provinces, followed by clade B_PA-II_ (detected in the provinces of Panama, Colon, Los Santos, Chiriquí and Comarca Ngöbe Bugle), clade B_PA-III_ (detected in the provinces of Panama, Colón, Coclé and Chiriquí) and clade B_PA-IV_ (detected in the provinces of Panama, Colon and Coclé) (Fig. 4). The root location for all major Panamanian clades was most probably traced to the districts of Panama and San Miguelito in the east of Panama province (posterior root state probability>0.80) (Table S3), that were also pointed as the major hub of dissemination of all viral clades. The districts of Arraiján and La Chorrera, in the west of Panama province, seem to be the main secondary hubs of dissemination, sending viruses mainly to the neighboring districts of Colón, Antón and also back to east of Panama province.

### Demographic History of Major HIV-1 Subtype B Panamanian Clades

**Figure 5:**
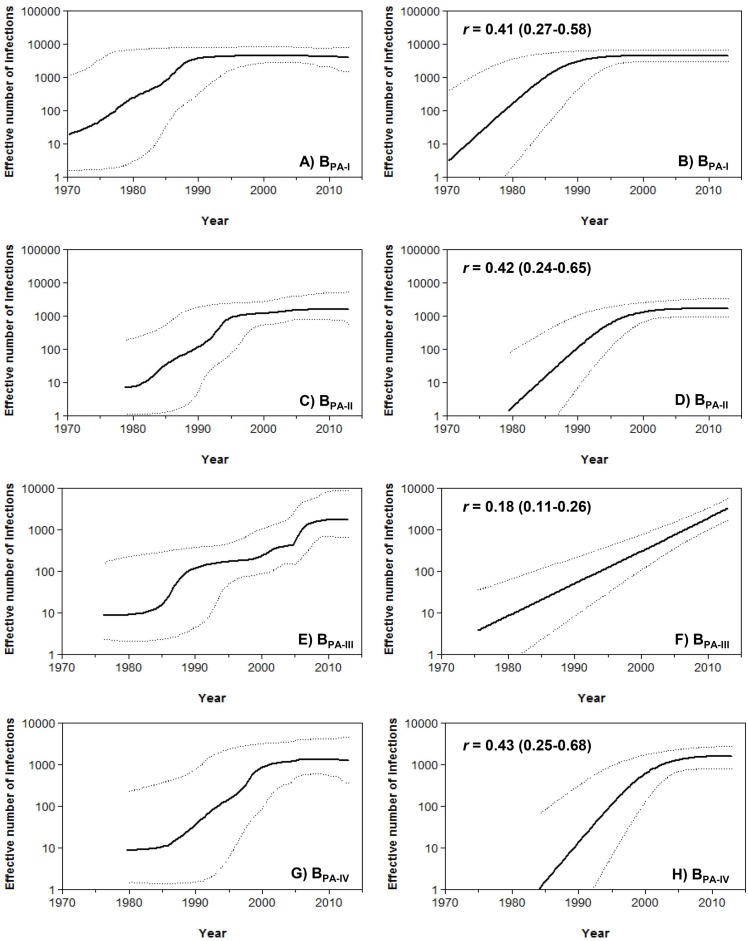
Demographic history of HIV-1 Panamanian clades of large size (B_PA-I_ to B_PA-IV_). Effective number of infections (*y*-axis; log_10_ scale) through time (x-axis; calendar years) estimated using Bayesian skyline (A, C, E and G) and logistic (B, D and F) or exponential (H) growth coalescent models are shown for each of the four HIV-1 Panamanian clades. Median estimates of the effective number of infections (solid line) and 95% HPD intervals of the estimates (dashed lines) are shown in each graphic. The median growth rate (with the corresponding 95% credibility interval in parenthesis) of each clade estimated under logistic or exponential growth model is indicated in the upper left corner.

Population dynamics of HIV-1 Panamanian clades of large size was next investigated. The Bayesian skyline plot (BSP) analyses suggest that clades B_PA-I_, B_PA-II_ and B_PA-IV_ experienced an initial phase of exponential growth followed by a decline in growth rate in the early 1990s, the middle 1990s and the early 2000s, respectively (Fig. 5). The clade B_PA-III_ seems to have experienced a more complex dynamics characterized by two phases of rapid expansion (1985–1990 and 2000–2010) interleaved by periods of slow growth (Fig. 5). To estimate the mean epidemic growth rate of the major Panamanian clades, log ML for the logistic, exponential and expansion growth models were calculated using both PS and SS methods. The best fit demographic model was the logistic for clades B_PA-I_, B_PA-II_ and B_PA-IV_ (log BF>5) and the exponential for clade B_PA-III_ (log BF>5) (Table S4). The overall time-scale and demographic pattern obtained from both BSP and logistic/exponential growth coalescent tree priors were very similar and the mean estimated growth rate of clades B_PA-I_, B_PA-II_ and B_PA-IV_ (∼0.4 year^−1^) was about two times higher than that estimated for the clade B_PA-III_ (∼0.2 year^−1^) (Fig. 5).

## Discussion

The HIV-1 subtype B is the most prevalent clade in the Americas. In this study we investigated the origin of the HIV-1 subtype B epidemic in Panama based on the largest number of viral sequences from this country analyzed up to date. Our results support a scenario of multiple (*n*>100) subtype B introductions into Panama. Most Panamanian subtype B viruses (94.5%) belong to the B_PANDEMIC_ clade, a viral strain originated in the US and disseminated worldwide [21], as was described in other Central American countries [17]. We also detected, however, a minor fraction (5.5%) of Panamanian subtype B viruses belonging to the most basal B_CAR_ clades of Caribbean origin [21]. It was previously proposed that some subtype B outbreaks in South America were directly seeded by the Caribbean epidemic [42]; but this is the first demonstration of the circulation of non-pandemic subtype B Caribbean clades in Central America. Notably, the first reported Panamanian AIDS case was an Haitian woman diagnosed in September 1984 [43], supporting a longstanding presence of viruses of Caribbean origin in Panama. Our data also suggests that B_CAR_ clades are mainly disseminated in Panama by heterosexual transmission, as was also reported in the Caribbean [44]; whereas the B_PANDEMIC_ clade is disseminated through both heterosexual and homosexual/bisexual networks.

A previous study analyzed the genetic diversity of 133 HIV-1 subtype B *pol* sequences from Panama and identified five country-specific monophyletic clusters of ≥5 sequences (designated as B-PA1 to B-PA5) that comprised nearly two thirds of Panamanian HIV-1 subtype B viruses [19]. Our phylogeographic analysis identifies 12 country-specific subtype B clades of >10 sequences (designated as B_PA-I_ to B_PA-XII_), that also comprised about two thirds of all Panamanian sequences here analyzed. Ten Panamanian clusters branched within the B_PANDEMIC_ clade whereas the remaining two branched among the B_CAR_ lineages. The branching position of the subtype B Panamanian sequences included in both studies indicates the following clades correspondence: B-PA1 = B_PA-I_, B-PA2 = B_PA-III_, B-PA3+B-PA5 = B_PA-II_, and B-PA4 = B_PA-IV_. These results confirm the existence of multiple active HIV-1 subtype B transmission chains in the Panamanian population and reveal that the number of such independent local networks is much larger than previously estimated. It is possible that further sampling would show additional Panamanian clades.

A recent study suggests that the majority (∼60%) of the current HIV-1 subtype B cases in Central America resulted from the regional dissemination of a single viral lineage, here designated B_CAM_, that derived from the B_PANDEMIC_ clade [17]. This previous study, however, included a very low number of subtype B sequences from Panama (*n* = 37) and nearly all of those sequences (*n* = 36) branched outside the B_CAM_ clade. Our study revealed that only 0.4% of Panamanian sequences branched within the B_CAM_ clade and only 0.l% of the sequences from other Central American countries fell within major Panamanian clades (B_PA-I_–B_PA-XII_). Thus, the HIV-1 subtype B epidemic in Panama is characterized by the dissemination of local country-specific viral lineages, limited viral flux with other Central American countries, and no evidence of expansion of the major B_CAM_ clade. A more extensive sampling from other Central American countries including Belize, Costa Rica, Guatemala and Nicaragua, will be crucial to estimate the relative prevalence of country-specific clades and the B_CAM_ clade in the region.

Previous studies estimated that major HIV-1 subtype B Panamanian clades started to spread not earlier than the middle 1980s [19], [23]. According to our estimations, however, HIV-1 subtype B Panamanian clades of large size (B_PA-I_ to B_PA-IV_) probably originated between the early and the middle 1970s, whereas clades of medium size (B_PA-V_ to B_PA-XII_) arise between the late 1970s and the early 1980s. The mean T_MRCA_ of large sized Panamanian clades here obtained (1973–1975) coincides with those estimated for other Central American clades (1966–1976) [17], and were also close to the estimated origin of the B_PANDEMIC_ clade in the US (1968–1969) and the B_CAR_ lineages in Haiti (1964–1966) [21], [42], [45], [46]. Thus, these results suggest that the HIV-1 subtype B epidemics in Panama and other Central American countries started shortly after (5–10 years) the emergence of subtype B epidemics in Haiti and the US.

The Panamanian clade B_PA-I_ was the earliest and most widely disseminated viral lineage being detected from the easternmost province (Darién) to the westernmost provinces (Bocas del Toro and Chiriquí); whereas, the other three major clades (B_PA-II_, B_PA-III_ and B_PA-IV_) were mainly detected in the central provinces (Panama, Coclé, Colón, and Los Santos).

Our findings suggest that the main hubs of dissemination of all subtype B Panamanian clades of large size are the districts of Panama (which includes the Panama city) and San Miguelito located in the eastern Panama province. These districts comprises about 70% of the sequences included in the major subtype B Panamanian clades (B_PA-I_ to B_PA-IV_) and also concentrate about 50% of the total number of HIV positive individuals diagnosed in the country between 1984 and 2010 [24]. Another densely populated area with a high incidence rate of HIV infections, the districts of Arraiján and La Chorrera in the western Panama province [24], was pointed as an important secondary hub of dissemination of major subtype B Panamanian clades. Some of the viral migrations originated in that region, however, were associated to ancestral nodes with low location probability supports (<0.60) (Fig. 4) and should be interpreted with caution.

Our demographic analysis indicates that Panamanian clades B_PA-I_, B_PA-II_ and B_PA-IV_ followed a roughly similar growth pattern characterized by a rapid initial dissemination phase after which the epidemic growth rate started to slow-down. The precise time of stabilization of the growth rate varied from the early 1990s for clade B_PA-I_ to the early 2000s for clade B_PA-IV_. The clade B_PA-III_ seems to have experienced a more complex population dynamic characterized by two phases of rapid expansion (1985–1990 and 2000–2010) interleaved by periods of slow growth. Interestingly, the most recent expansion phase of clade B_PA-III_ coincides with a higher frequency of newly diagnosed subjects (<1 year of documented HIV-1 infection) within this clade (50%) when compared with the other major Panamanian clades (32–35%). These observations suggest that clade B_PA-III_ may have been spreading among recently infected people more efficiently than others clades and that we could expect an increase in the relative prevalence of clade B_PA-III_ in the following years in Panama.

The mean estimated growth rate of clades B_PA-I_, B_PA-II_ and B_PA-IV_ (∼0.4 year^−1^) was about two times higher than that estimated for the clade B_PA-III_ (∼0.2 year^−1^); but much lower than those previously estimated for a number of country-specific subtype B clades circulating in Cuba (∼0.7–1.6 year^−1^) [47], Italy (∼1.5 year^−1^) [48], China/Hong Kong (∼0.9–1.3 year^−1^) [49] and the United Kingdom (∼0.5–1.1 year^−1^) [50]. The HIV-1 subtype B clades described previously were mainly or exclusively restricted to populations of men-having-sex-with-men (MSM) [47]–[50]; while females comprise a significant fraction (34–40%) of Panamanian clades B_PA-I_, B_PA-II_ and B_PA-IV_. Thus, differences in epidemic growth rates among subtype B clades may be explained by the epidemiological characteristics of the viral transmission networks. This hypothesis, however, does not explain the low expansion rate of clade B_PA-III_ that contains a much higher proportion of MSM and lower proportion of females than the other major Panamanian clades. It is possible that the exponential growth model used is too simple to capture the complex population dynamic of clade B_PA-III_ and that the actual expansion rate of this clade is higher than estimated.

In summary, this study demonstrates that the HIV-1 subtype B epidemic in Panama is mainly driven by dissemination of multiple subtype B founder viral strains that were probably introduced from the Caribbean and other American countries between the early 1970s and the early 1980s. Circulation of Panamanian subtype B clades remained mainly restricted to this country, thus indicating that Panama is not a major source of subtype B in Central America. Although Panamanian subtype B clades of large size emerged in the same location (western districts of Panama province) and around the same time (1970–1975), the subsequent spatial dissemination and population growth patterns were different across clades. This study offers important insights into understanding the dissemination dynamics of the HIV-1 subtype B epidemic in Panama and the Central American region.

## Supporting Information

Figure S1 **Geographic distribution of HIV-1 subtype B Panamanian sequences used in this study.** Map of Panama indicating the number of sequences located in each of the provinces and native territories of Comarcas Kuna Yala and Ngobe Bugle.
(TIFF)

Table S1 **Epidemiological information of subjects infected with HIV-1 Panamanian clades BPA-V to BPA-XII.**
(PDF)

Table S2 **Evolutionary rate of major HIV-1 subtype B Panamanian clades.**
(PDF)

Table S3 **Posterior probability distributions for the root location of the major HIV-1 subtype B Panamanian clades.**
(PDF)

Table S4 **Best fit demographic model for major HIV-1 subtype B Panamanian clades.**
(PDF)
