# Supplementary figures and images for: Human Immunodeficiency Virus Type 1 (HIV-1) Subtype B Epidemic in Panama Is Mainly Driven by Dissemination of Country-Specific Clades
Source: PLoS One. 2014 Apr 18;9(4):e95360. doi: 10.1371/journal.pone.0095360 (PMC3991702; doi:10.1371/journal.pone.0095360)

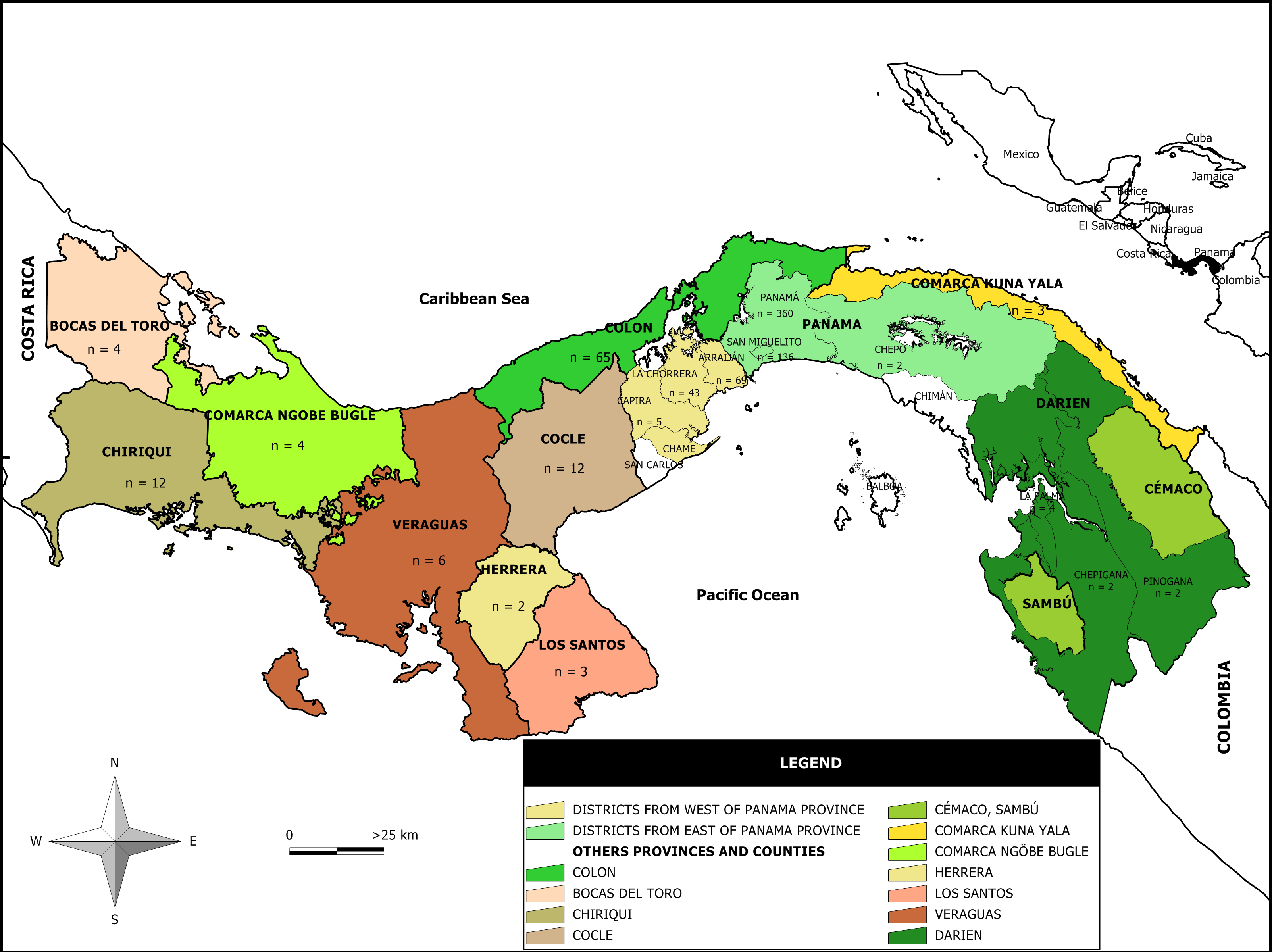

Supplement: Figure S1 — Geographic distribution of HIV-1 subtype B Panamanian sequences used in this study. Map of Panama indicating the number of sequences located in each of the provinces and native territories of Comarcas Kuna Yala and Ngobe Bugle. (TIFF) [file pone.0095360.s001.tif]
