# Supplementary material for: Human Immunodeficiency Virus Type 1 (HIV-1) Subtype B Epidemic in Panama Is Mainly Driven by Dissemination of Country-Specific Clades
Source: PLoS One. 2014 Apr 18;9(4):e95360. doi: 10.1371/journal.pone.0095360 (PMC3991702; doi:10.1371/journal.pone.0095360)
Supplement: Table S1 — Epidemiological information of subjects infected with HIV-1 Panamanian clades BPA-V to BPA-XII. (PDF) [file pone.0095360.s002.pdf]

Table S1. Epidemiological information of subjects infected with HIV-1 Panamanian clades B_{PA-V} to B_{PA-XII}.

	**B_{PA-V} (n=23)**	**B_{PA-VI} (n=22)**	**B_{PA-VII} (n=17)**	**$B_{PA-VIII}$ (n=17)**	**B_{PA-IX} (n=12)**	**B_{PA-X} (n=10)**	**B_{PA-XI} (n=10)**	**B_{PA-XII} (n=9)**	**B_{PA-}Non-clustered (n=52)**
**Sampling interval (years)**	2008-2013	2008-2013	2008-2013	2009-2013	2009-2012	2009-2013	2007-2013	2011-2013	2008-2013
**HIV diagnosis**									
1987-1994	1 (4.3)	-	1 (5.9)	1 (5.9)	-	-	-	-	1 (1.9)
1995-1999	1 (4.3)	2 (9.1)	-	3 (17.6)	2 (16.7)	-	-	-	2 (3.8)
2000-2004	3 (13.0)	9 (40.9)	9 (52.9)	4 (23.5)	2 (16.7)	3 (30)	5 (50)	3 (33.3)	11 (21.2)
2005-2009	11 (47.8)	6 (27.3)	6 (35.3)	5 (29.4)	5 (41.6)	5 (50)	5 (50)	1 (11.1)	18 (34.6)
2010-2013	6 (26.1)	5 (22.7)	-	4 (23.5)	2 (16.7)	2 (20)	-	5 (55.5)	20 (38.5)
unknown	1 (4.3)	-	1 (5.9)	-	1 (8.3)	-	-	-	-
**Sex**									
Male	17 (73.9)	14 (63.6)	10 (58.8)	6 (35.3)	10 (83.3)	4 (40.0)	7 (70.0)	8 (88.9)	36 (69.2)
Female	6 (26.1)	8 (36.4)	7 (41.2)	11 (64.7)	2 (16.7)	6 (60.0)	3 (30.0)	1 (11.1)	16 (30.8)
**Mode of Transmission**									
Homosexual / Bisexual	2 (8.7)	2 (9.1)	-	-	1 (8.3)	-	-	2 (22.2)	10 (19.2)
Heterosexual	4 (17.4)	6 (27.3)	5 (29.4)	12 (70.6)	3 (25.0)	7 (70.0)	3 (30.0)	2 (22.2)	18 (34.6)
Mother to Child	4 (17.4)	4 (18.2)	3 (17.6)	1 (5.9)	-	-	3 (30.0)	-	3 (5.8)
Others (blood products)	-	-	-	-	-	-	-	-	1 (1.9)
Unknown	13 (56.5)	10 (45.5)	9 (52.9)	4 (23.5)	8 (66.7)	3 (30.0)	4 (40.0)	5 (55.6)	20 (38.5)
**Age Group (years)**									
< 14	4 (17.4)	4 (18.2)	2 (11.8)	2 (11.8)	-	-	3 (30.0)	-	3 (5.8)
15-24	4 (17.4)	5 (22.7)	1 (5.9)	2 (11.8)	1 (8.3)	2 (20.0)	-	1 (11.1)	11 (21.2)
25-34	6 (26.1)	3 (13.6)	-	7 (41.2)	6 (50.0)	5 (50.0)	2 (20.0)	3 (33.3)	13 (25.0)
35-44	4 (17.4)	8 (36.4)	4 (23.5)	3 (17.6)	5 (41.7)	2 (20.0)	5 (50.0)	2 (22.2)	14 (26.9)
45-54	3 (13.0)	2 (9.1)	9 (52.9)	2 (11.8)	-	1 (10.0)	-	2 (22.2)	6 (11.5)
> 55	2 (8.7)	-	1 (5.9)	1 (5.9)	-	-	-	1 (11.1)	5 (9.6)
**Clinical Condition**									
Asyntomatic / acute	13 (56.5)	11 (50.0)	6 (35.3)	8 (47.1)	6 (50.0)	5 (50.0)	6 (60.0)	5 (55.6)	36 (69.2)
AIDS	5 (21.7)	6 (27.3)	8 (47.1)	7 (41.2)	3 (25.0)	1 (10.0)	3 (30.0)	4 (44.4)	10 (19.2)
Unknown	5 (21.7)	5 (22.7)	3 (17.6)	2 (11.8)	3 (25.0)	4 (40.0)	1 (10.0)	-	9 (17.3)
**Geographic location**									
East of Panama Province	15 (65.2)	13 (59.1)	5 (29.4)	9 (52.9)	6 (50.0)	2 (20.0)	5 (50.0)	7 (77.8)	34 (65.4)
West of Panama Province	2 (8.7)	6 (27.3)	2 (11.8)	4 (23.5)	4 (33.3)	-	-	-	3 (5.8)
Colon Province	-	-	9 (52.9)	2 (11.8)	1 (8.3)	4 (40.0)	2 (20.0)	-	7 (13.5)
Others provinces	6 (26.1)	3 (13.6)	1 (5.9)	1 (5.9)	1 (8.3)	4 (40.0)	3 (30.0)	1 (11.1)	5 (9.6)
Unknown	-	-	-	1 (5.9)	-	-	-	-	3 (5.8)

Data are No. (%). Most Panamanian subtype B clades belong to the $B_{PANDEMIC}$ lineage, with exception of clades $B_{PA-VIII}$ and B_{PA-IX} that belong to the B_{CAR} lineages.
