# Supplementary material for: Human Immunodeficiency Virus Type 1 (HIV-1) Subtype B Epidemic in Panama Is Mainly Driven by Dissemination of Country-Specific Clades
Source: PLoS One. 2014 Apr 18;9(4):e95360. doi: 10.1371/journal.pone.0095360 (PMC3991702; doi:10.1371/journal.pone.0095360)
Supplement: Table S2 — Evolutionary rate of major HIV-1 subtype B Panamanian clades. (PDF) [file pone.0095360.s003.pdf]

Table S2. Evolutionary rate of major HIV-1 subtype Panamanian clades.

Clade	Dataset	Substitution rate	Coefficient of variation
B_{PA-I}	$B_{PA-I/XII}$	$1.9x10^{-3}$ ($1.6x10^{-3}$-$2.3x10^{-3}$)	0.29 (0.26-0.33)
	B_{PA-I}	$1.7x10^{-3}$ ($1.1x10^{-3}$-$2.3x10^{-3}$)	0.29 (0.23-0.35)
B_{PA-II}	$B_{PA-I/XII}$	$1.9x10^{-3}$ ($1.6x10^{-3}$-$2.3x10^{-3}$)	0.29 (0.26-0.33)
	B_{PA-II}	$1.7x10^{-3}$ ($1.2x10^{-3}$-$2.3x10^{-3}$)	0.31 (0.22-0.40)
B_{PA-III}	$B_{PA-I/XII}$	$1.9x10^{-3}$ ($1.6x10^{-3}$-$2.3x10^{-3}$)	0.29 (0.26-0.33)
	B_{PA-III}	$1.6x10^{-3}$ ($1.0x10^{-3}$-$2.1x10^{-3}$)	0.21 (0.04-0.35)
B_{PA-IV}	$B_{PA-I/XII}$	$1.9x10^{-3}$ ($1.6x10^{-3}$-$2.3x10^{-3}$)	0.29 (0.26-0.33)
	B_{PA-IV}	$1.8x10^{-3}$ ($1.0x10^{-3}$-$2.6x10^{-3}$)	0.32 (0.20-0.44)

Substitution rate (subst./site/year) and coefficient of rate variation estimated from the combined ($B_{PA-I/XII}$) and the single clade (B_{PA-I}, B_{PA-II}, B_{PA-III}, and B_{PA-IV}) datasets.
