# Supplementary material for: Human Immunodeficiency Virus Type 1 (HIV-1) Subtype B Epidemic in Panama Is Mainly Driven by Dissemination of Country-Specific Clades
Source: PLoS One. 2014 Apr 18;9(4):e95360. doi: 10.1371/journal.pone.0095360 (PMC3991702; doi:10.1371/journal.pone.0095360)
Supplement: Table S3 — Posterior probability distributions for the root location of the major HIV-1 subtype B Panamanian clades. (PDF) [file pone.0095360.s004.pdf]

Table S3. Posterior probability distributions for the root location of the major HIV-1 subtype B Panamanian clades.

Clade	East Panama province [a]	West Panama province [b]	Other provinces
B_{PA-I}	0.99	0.01	0
B_{PA-II}	0.99	0.01	0
B_{PA-III}	0.97	0.03	0
B_{PA-IV}	0.82	0.17	0.01

[a] Districts of Panama and San Miguelito. [b] Districts of Arraiján and La Chorrera.
