# Supplementary material for: Human Immunodeficiency Virus Type 1 (HIV-1) Subtype B Epidemic in Panama Is Mainly Driven by Dissemination of Country-Specific Clades
Source: PLoS One. 2014 Apr 18;9(4):e95360. doi: 10.1371/journal.pone.0095360 (PMC3991702; doi:10.1371/journal.pone.0095360)
Supplement: Table S4 — Best fit demographic model for major HIV-1 subtype B Panamanian clades. (PDF) [file pone.0095360.s005.pdf]

Table S3. Best fit demographic model for major HIV-1 Panamanian clades.

Clade	Model	PS Log ML	Models compared	Log BF	SS Log ML	Models compared	Log BF
B_{PA-I}	**Log**	**-21712.66**	-	-	**-21720.00**	-	-
	Expo	-21789.31	Log/Expo	76.7	-21794.16	Log/Expo	74.2
	Expa	-21791.31	Log/Expa	78.7	-21795.71	Log/Expa	75.7
B_{PA-II}	**Log**	**-10904.10**	-	-	**-10908.13**	-	-
	Expo	-10925.20	Log/Expo	21.1	-10930.91	Log/Expo	22.8
	Expa	-10931.61	Log/Expa	27.5	10938.61	Log/Expa	30.5
B_{PA-III}	**Expo**	**-7839.35**	-	-	**-7839.54**	-	-
	Expa	-7845.16	Expo/ Expa	5.8	-7845.52	Expo/ Expa	6.0
	Log	-7846.01	Expo/Log	6.7	-7846.20	Expo/Log	6.7
B_{PA-IV}	**Log**	**-7319.12**	-	-	**-7321.12**	-	-
	Expo	-7329.97	Log/Expo	10.8	-7326.78	Log/Expo	5.7
	Expa	-7333.95	Log/Expa	14.8	-7331.78	Log/Expa	10.7

Log marginal likelihood (ML) estimates for the logistic (Log), exponential (Expo) and expansion (Expa) growth demographic models obtained using the path sampling (PS) and stepping-stone sampling (SS) methods. The Log Bayes factor (BF) is the difference of the Log ML between of alternative (H1) and null (H0) models (H1/H0). Log BFs > 3 indicate that model H1 is more strongly supported by the data than model H0. The best fit demographic models are marked in bold.
